# Ventriculoperitoneal shunt is associated with increased cerebrospinal fluid protein level in HIV-infected cryptococcal meningitis patients

**DOI:** 10.1186/s12879-022-07286-6

**Published:** 2022-03-26

**Authors:** Ran Tao, Lijun Xu, Yongzheng Guo, Xiaoke Xu, Jiesheng Zheng, Biao Zhu

**Affiliations:** 1 National Clinical Research Center for Infectious Diseases, the First Affiliated Hospital, College of Medicine, Zhejiang University, Hangzhou, China; 2 The State Key Laboratory for Diagnosis and Treatment of Infectious Diseases, the First Affiliated Hospital, College of Medicine, Zhejiang University, Hangzhou, China; 3 Department of Neurosurgery, The First Affiliated Hospital, College of Medicine, Zhejiang University, Hangzhou, China

**Keywords:** Ventriculoperitoneal shunt, Human immunodeficiency virus, Cryptococcal meningitis, Survival, Cerebrospinal fluid

## Abstract

**Background:**

The impact of ventriculoperitoneal shunt on cerebrospinal fluid (CSF) biochemical profiles in HIV-associated cryptococcal meningitis (HCM) patients remains unclear.

**Methods:**

Twenty-nine HCM patients who underwent ventriculoperitoneal shunt (the VPS group) and 57 HCM patients who did not undergo ventriculoperitoneal shunt (the non-VPS group) were enrolled in this propensity score matching analysis. Demographic characteristics, symptoms, CSF biochemical profiles, and adverse events were compared between the two groups. The Kaplan–Meier method was used to analyze the survival rate. Univariate and multivariate logistic regression analyses were performed to identify the risk factors for increased CSF protein levels.

**Results:**

After 24 weeks of treatment, the intracranial pressure was significantly lower in the VPS group than in the non-VPS group (mmH_2_O; 155.0 [120.0–190.0] vs. 200.0 [142.5–290.0]; P = 0.025), and the rate of neuroimaging improvement was significantly higher in the VPS group (16/17 [94.1%] vs. 2/10 [20%]; P < 0.001). Furthermore, the 24-week cumulative survival rates were also significantly higher in the VPS group (96.6% vs. 83.5%, P = 0.025). Notably, the CSF protein levels were higher in the VPS group than in the non-VPS group at each examination time, and the CSF glucose was lower in the VPS group than in the non-VPS group even at the 12-week follow-up. In the multivariate analysis, we found that VPS placement was an independent risk factor for increased CSF protein (odds ratio [OR]: 27.8, 95% confidence interval [95% CI] 2.2–348.7; P = 0.010).

**Conclusions:**

VPS decreased the intracranial pressure, improved neuroimaging radiology and reduced the 24-week mortality in HCM patients. However, VPS significantly altered the CSF profiles, which could lead to misdiagnosis of tuberculous meningitis and some of them were diagnosed with immune reconstitution inflammatory syndrome. Physicians should be aware of these changes in the CSF profiles of patients with HCM undergoing VPS.

## Background

HIV-infected patients are susceptible to *Cryptococcus*, and cryptococcal meningitis is a life-threatening central nervous system infectious disease caused by *Cryptococcus*. Importantly, high intracranial pressure (HICP) can occur in approximately 50% of patients, leading to increased mortality in patients with HCM [1–4]. Thus, HICP control is a critical determinant of HCM patient mortality [5].

Daily lumbar puncture and the placement of ventriculoperitoneal shunts (VPSs) are important management strategies for HICP patients. However, the placement of VPSs in HIV-infected patients is debatable. Some studies have indicated that VPS placement in immunosuppressed patients may lead to shunt infection, blockage of the shunt device owing to the high fungal load, and peritoneal *Cryptococcus* seeding by draining *Cryptococcus* into the abdominal cavity [6–8]. Meanwhile, other studies have demonstrated that VPS placement can rapidly relieve symptoms and improve the prognosis of HICP patients with rare postoperative infections. In addition, VPS placement could decrease the excess volume of cerebrospinal fluid (CSF) and the fungal polysaccharide load in the ventricles [9–12]. VPS is more reliable and stable than lumber puncture and can maintain long-term shunt effects [6].

To further study the safety and efficacy of VPS placement in HCM patients and evaluate the effects of VPS placement on the CSF biochemical profiles of HCM patients, we conducted this study. In this study, we compared the demographic characteristics, symptoms, and CSF chemical profiles between HCM patients who underwent VPS placement and HCM patients who did not undergo VPS placement.

## Methods

### Study cohort and patient enrollment

**Fig. 1:**
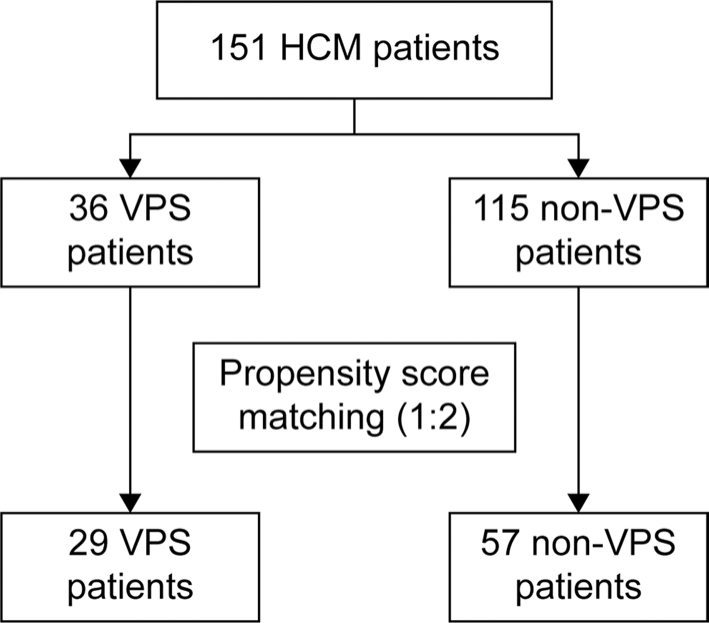
Patient selection flowchart. *HCM* human immunodeficiency virus-associated cryptococcal meningitis, *VPS* ventriculoperitoneal shunt

Between January 2011 and December 2019, 151 HCM patients from the First Affiliated Hospital of Zhejiang University were eligible for this retrospective cohort study. Of these patients, 36 (23.8%) underwent VPS placement (the VPS group), and 115 (76.2%) did not undergo VPS placement (the non-VPS group). Propensity score matching for age, sex, body mass index (BMI), positive India ink staining of the CSF, positive *Cryptococcus* cultures, initial CSF profiles (intracranial pressure [ICP], CSF glucose levels, CSF protein levels, and CSF white blood cell [WBC] counts), and routine blood test results (C-reactive protein levels, WBC counts, hemoglobin levels, platelet counts, and albumin levels) at admission was used to match the patients at a ratio of 1:2. Thirty-five patients in the VPS group and 62 in the non-VPS group were initially selected for the study. Finally, 29 patients who accepted VPS placement and 57 who did not accept VPS placement were enrolled in this study after 11 repeated cases were excluded. The patient selection process is illustrated in Fig. 1.

### Diagnostic criteria

Cryptococcal meningitis (CM) was diagnosed if at least one of the following criteria was present in our previous study [13]: (i) a positive CSF culture for *Cryptococcus neoformans*; (ii) positive India ink staining of cryptococci in centrifuged CSF sediment; (iii) encapsulated yeast cells in brain tissue, as observed using Gomori-methenamine silver and/or periodic acid–Schiff staining; and (iv) a positive CSF sample cryptococcal antigen test.

### Therapeutic approaches

All patients were treated with antifungal therapy immediately after diagnosis with cryptococcal meningitis. The induction regimens for HCM patients included amphotericin B (AmB) (0.7–1.0 mg/kg/day) plus 5-formylcytosine (5FC) (100 mg/kg/day) and fluconazole (1200 mg/day) ± 5FC (100 mg/kg/day) according to previous recommendations [14]. Routine lumbar puncture was performed weekly to monitor CSF profiles and ICP or in response to HICP symptoms (such as headache, vomiting, and dizziness). Mannitol and furosemide were administered to patients with an ICP of 200–300 mmH_2_O [15], whereas VPS placement was performed in patients with an ICP of ≥ 300 mmH_2_O who were willing to undergo operation to control the opening pressure, while daily lumbar puncture was performed in those not willing to undergo operation. Highly active antiretroviral therapy (HAART) was initiated after 4 weeks of antifungal therapy. The patients were followed up for 24 weeks and then discharged.

### Laboratory tests

Routine blood tests, biochemical tests, and CSF assays (opening pressure, WBC counts, glucose levels, protein levels, India ink staining, and cultures) were performed upon the first admission and at subsequent follow-up visits. Neuroimaging findings were independently assessed by two experienced neuroradiologists in a blinded manner.

### Follow-up and data collection

Patient data (BMI, blood test results, imaging examination results, treatments received, and follow-ups) were obtained from the hospital’s electronic medical record system (HEMRS). Week 0 was defined as the time of patient admission, and Week 1 was the first week after VPS placement for the VPS group and the first week of antifungal treatment for the non-VPS group. Patients were followed up for 24 weeks. Data from weeks 0 (W0), 1 (W1), 2 (W2), 4 (W4), 12 (W12), and 24 (W24) were analyzed.

### Statistical analyses

Continuous normally distributed variables are presented as the means ± standard deviations. Continuous nonnormally distributed variables are presented as the medians (interquartile ranges). Categorical variables are presented as the numbers of cases (percentages). Continuous variables were compared using Student’s t test or the Mann–Whitney U test, whereas categorical variables were compared using the χ^2^ test or Fisher’s exact test. CSF profile data from W24 and W0 in the VPS and non-VPS groups were compared using a paired t test or Wilcoxon test. Survival was analyzed using the Kaplan–Meier method. Risk factors for increased CSF protein levels were analyzed using logistic regression, and a univariate analysis of covariates was performed. Covariates with P < 0.2 in the univariate analysis were included in the multivariate analysis using the forward (stepwise ratio) method. Statistical analyses were performed using IBM SPSS 23.0 (IBM Corp., Armonk, NY, USA) and GraphPad Prism (version 8.0; GraphPad Software, La Jolla, CA, USA). A P value of < 0.05 (two tailed) was considered statistically significant.

### Ethics approval

This study protocol was conducted in accordance with the 1975 Helsinki Declaration and was approved by the Ethics Committee of the First Affiliated Hospital, College of Medicine, Zhejiang University (Hangzhou, China) (No. 2017-688). All data analyzed were anonymous. The ethics committee waived the requirement of written informed consent for participation.

## Results

### Baseline characteristics

There were 86 patients (including 29 in the VPS group and 57 in the non-VPS group) enrolled in the present study. Of these patients, 78/86 (90.7%) were male, and 8/86 (9.3%) were female. The mean age was 34.5 ± 8.4 years old, and the BMI was 20.5 ± 2.8 kg/m^2^. Positive cryptococcus blood cultures totalled 9/29 (31.0%) in the VPS group and 19/57 (33.3%) in the non-VPS group (P = 0.830). No significant difference in the initial ICP was observed between the VPS and non-VPS groups (mmH_2_O; 335.0 [252.5–416.3] vs. 300.00 [195.0–400.0]; P = 0.446). The initial CSF protein level and initial CD4 count were similar between the two groups (CSF protein [g/L]: 0.6 [0.4–1.0] vs. 0.6 [0.4–0.9], P = 0.980; CD4 count [/mL]: 11.0 [6.0–24.8] vs. 27.5 [13.0–41.8]; P = 0.386). The most common symptoms were headache (59/86, 68.6%), fever (51/86, 59.3%), vomiting (28/86, 32.6%), and dizziness (13/86, 15.1%). There was no difference in the occurrence of fever (P = 0.578), headache (P = 0.587), vomiting (P = 0.083), or dizziness (P = 0.096) between the two groups (Table 1). However, patients in the VPS group had a significantly higher incidence of seizures than those in the non-VPS group (6/29 [20.7%] vs. 2/57 [3.5%]; P = 0.010). The demographic characteristics and laboratory test results are shown in Table 1.

**Table 1:** Differences in the clinical features of patients with HCM between the VPS and Non-VPS groups

Factors	Non-VPS group (n = 57)	VPS group (n = 29)	P-value
Sex (male), n (%)	52 (91.2%)	26 (89.7%)	0.812
Age (years)	34.3 ± 5.0	34.5 ± 9.0	0.438
BMI	19.95 ± 2.9	20.58 ± 2.9	0.309
Interval from symptom onset to the initiation of anticryptococcal therapy (days)	10.0 (1.5–27.0)	10.0 (5.5–22.5)	0.985
Blood cryptococcus culture positivity, n (%)	19 (33.3%)	9 (31.0%)	0.830
Clinical manifestations, n (%)			
Fever	35 (61.4%)	16 (55.2%)	0.578
Headache	38 (66.7%)	21 (72.4%)	0.587
Dizziness	6 (10.5%)	7 (24.1%)	0.096
Seizures	2 (3.5%)	6 (20.7%)	0.010
Vomiting	15 (26.3%)	13 (44.8%)	0.083
Vision loss	3 (5.3%)	2 (6.9%)	0.76
Hearing loss	2 (3.5%)	2 (6.9%)	0.481
Disturbance of consciousness	7 (12.3%)	2 (6.9%)	0.441
First CSF assay			
ICP (mmH_2_O)	300.0 (195.0–400.0)	335.0 (252.5–416.3)	0.446
Glucose (mmol/L)	2.5 (1.9–2.9)	2.4 (1.7–3.3)	0.866
Total protein (g/L)	0.6 (0.4–0.9)	0.6 (0.4–1.0)	0.98
WBC count (× 10^6^/L)	10.0 (2.8–34.0)	3.0 (0–20.0)	0.084
Chlorine (mmol/L)	116.4 ± 6.3	119.7 ± 5.6	0.192
Cryptococcus neoformans count (/HPF)	3.0 (0.8–28.5)	3.0 (0.0–27.0)	0.213
Positive India ink staining, n (%)	47 (82.5%)	25 (86.2%)	0.656
Positive Cryptococcus culture, n (%)	48 (84.2%)	27 (93.1%)	0.243
Blood test results			
C-reactive protein (mg/L)	6.05 (3.2–24. 0)	7.8 (3.4–18.8)	0.82
WBC (× 10^9^/L)	5.0 (3.3–6.7)	5.8 (4.4–7.8)	0.084
Hemoglobin (g/L)	124.4 ± 27.5	123.3 ± 21.8	0.853
Platelet (× 10^9^/L)	204. 9 ± 77.1	218.2 ± 116.1	0.526
Albumin (g/L)	38.4 (33.4–42.9)	39.1 (37.2–40.4)	0.594
CD4 (/mL)	27.5 (13.0–41.8)	11.0 (6.0–24.8)	0.386

*CM* cryptococcal meningitis, *CSF* cerebrospinal fluid, *HCM* HIV-associated cryptococcal meningitis, *HIV* human immunodeficiency virus, *HPF* high-power field, *ICP* intracranial pressure, *VPS* ventriculoperitoneal shunt, *WBC* white blood cell

### Changes in clinical symptoms and CSF profiles after 24 weeks of follow-up

The most common symptoms included headache (13/73, 17.8%) and fever (6/73, 8.2%) in all patients after 24 weeks of treatment. Patients in both groups reported no dizziness, hearing loss, or loss of consciousness after 24 weeks of treatment. Fever, headache, dizziness, and vomiting improved significantly in both groups after 24 weeks of treatment (Table 2).

**Table 2:** Changes in clinical symptoms and CSF profiles after 24 weeks in the groups

Factors	VPS group			Non-VPS group		
	Baseline-W0 (n = 29)	24 weeks of follow-up (n = 28)	P-value	Baseline-W0 (n = 57)	24 weeks of follow-up (n = 45)	P-value
Cerebrospinal fluid						
ICP (mmH_2_O)	335.0 (252.5–407.5)	155.0 (120.0–190.0)	0.001	300.0 (195.0–400.0)	200.0 (142.5–290.0)	0.394
Glucose (mmol/L)	2.4 (1.7–3.3)	2.4 (2.2–3.1)	0.868	2.5 (1.9–2.9)	2.8 (2.6–3.4)	0.072
Chlorine (mmol/L)	119.7 ± 5.6	120.2 ± 5.0	0.496	116.4 ± 6.3	121.0 ± 5.8	0.066
Total protein (g/L)	0.6 (0.4–1.0)	1.1 (0.6–1.6)	0.045	0.6 (0.4–0.9)	0.4 (0.3–0.7)	0.14
WBC count (× 10^6^/L)	3.0 (0–20.0)	10 (1.0–30.0)	0.641	10.0 (2.8–34.0)	15.0 (3.0–36.5)	0.433
Cryptococcus neoformans count (/HPF)	3.0 (0.0–27.0)	0 (0–1.0)	0.002	3.0 (0.8–28.5)	0 (0–1.0)	0.003
Clinical manifestations, n (%)						
Fever	16 (55.2%)	1 (3.6%)	< 0.001	35 (61.4%)	5 (11.1%)	< 0.001
Headache	21 (72.4%)	7 (25.0%)	< 0.001	38 (66.7%)	6 (13.3%)	< 0.001
Dizziness	7 (24.1%)	0 (0)	0.006	6 (10.5%)	0 (0)	0.025
Seizures	6 (20.7%)	2 (7.1%)	0.141	2 (3.5%)	0 (0)	0.204
Vomiting	13 (44.8%)	2 (7.1%)	0.001	15 (26.3%)	0 (0)	< 0.001
Vision loss	2 (6.9%)	0 (0)	0.157	3 (5.3%)	1 (2.2%)	0.432
Hearing loss	2 (6.9%)	0 (0)	0.157	2 (3.5%)	0 (0)	0.2
Disturbance of consciousness	2 (6.9%)	0 (0)	0.157	7 (12.3%)	0 (0)	0.014

*HCM* HIV-associated cryptococcal meningitis, *HPF* high-power field, *ICP* intracranial pressure, *VPS* ventriculoperitoneal shunt, *WBC* white blood cell

**Fig. 2:**
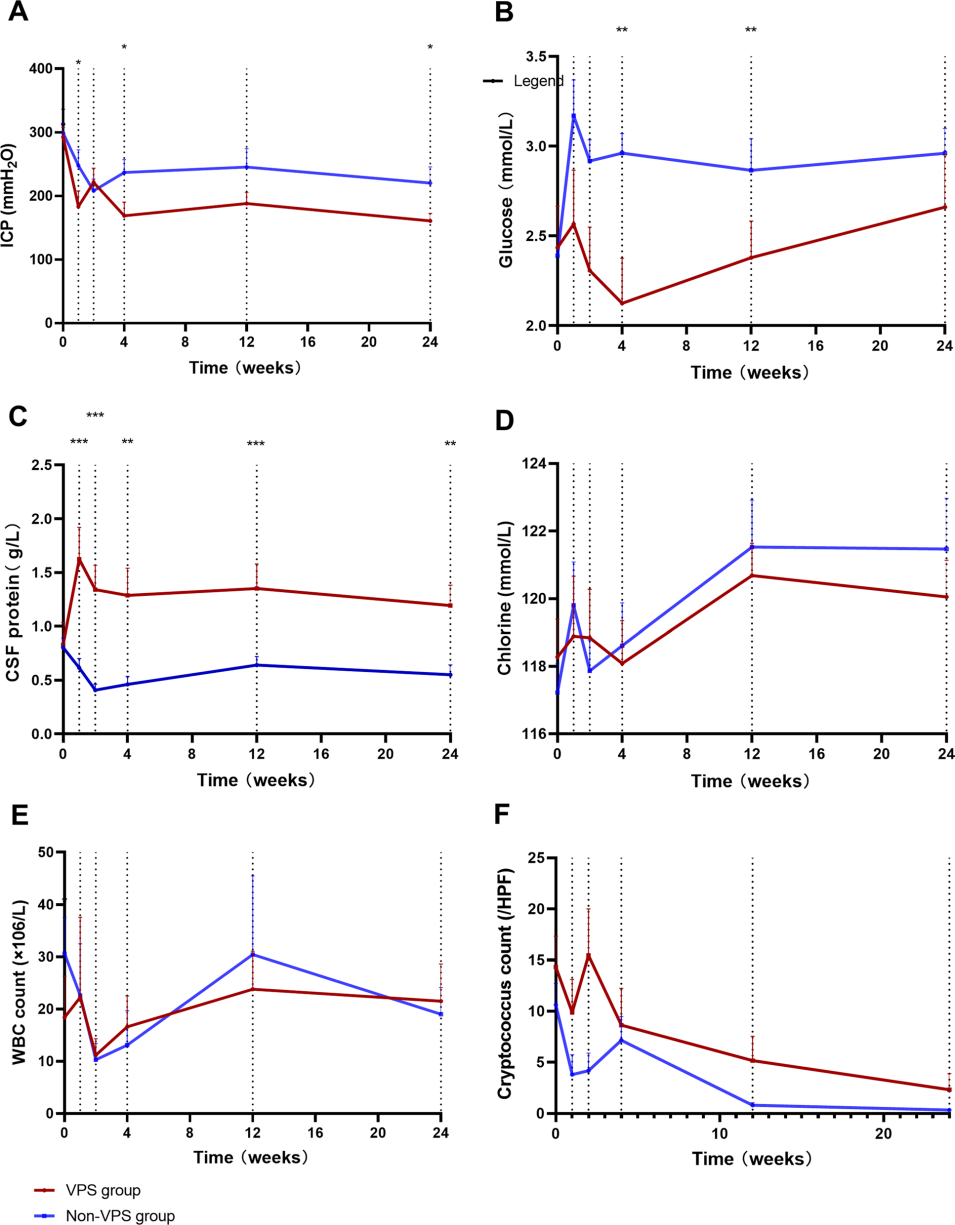
Comparison of CSF profiles and ICP values between the VPS and non-VPS groups (*P < 0.050, **P < 0.010, ***P < 0.001). *CSF* cerebrospinal fluid, *HPF* high-power field, *ICP* intracranial hypertension, *VPS* ventriculoperitoneal shunt, *WBC* white blood cell

In the VPS group, the ICP decreased rapidly 1 week after VPS placement (mmH_2_O; VPS group: 140.0 [101.3–167.5] vs. non-VPS group: 192.5 [152.5–327.5], P = 0.030) and then remained stably low. By W24, the ICP in the VPS group (155.0 [120.0–190.0] mmH_2_O) was significantly lower than that in the non-VPS group (mmH_2_O; 200.0 [142.5–290.0]) (P = 0.025) (Fig. 2A).

After VPS placement, the mean CSF glucose in the VPS group was persistently lower than that in the non-VPS group at each examination time. In particular, the nadir CSF glucose was 2.1 ± 0.87 mmol/L at week 4 in the VPS group, which was significantly lower than the value of 3.0 ± 0.46 mmol/L in the non-VPS group (P = 0.002). There was no significant difference in CSF glucose at week 24 (Fig. 2B).

The CSF protein was 0.6 (0.4–1.0) g/L at W0, 1.3 (0.9–1.7) g/L at W1, and 1.1 (0.6–1.6) g/L at W24 in the VPS group, whereas the CSF protein was 0.6 (0.4–0.9) g/L at W0, 0.5 (0.4–0.7) g/L at W1, and 0.4 (0.3–0.7) g/L at W24 in the non-VPS group (P = 0.98; P < 0.001; P = 0.002, respectively) (Fig. 2C). At W24, 17/19 (89.5%) patients in the VPS group and 7/16 (43.8%) in the non-VPS group had CSF protein levels ≥ 0.5 g/L (P = 0.004).

There was no significant difference in CSF chlorine, CSF WBC count and CSF cryptococcus count during the 24 week follow up between the two groups (Fig. 2D–F).

Risk factors for increased CSF protein levels ≥ 0.5 g/L at W24 were analyzed. In the unadjusted model, we found that VPS placement (odds ratio [OR]: 10.9, 95% confidence interval [95% CI] [1.9–64.0], P = 0.008), an increase in the CD4 count of > 100 cells/mL after 24 weeks (OR: 8.5, 95% CI [0.9–76.9], P = 0.058), a positive CSF culture (OR: 2.9, 95% CI [0.7–11.7], P = 0.127), and an initial CD4 count of > 20 cells/mL (OR: 0.3, 95% CI [0.1–1.4], P = 0.112) were risk factors for increased CSF protein levels. However, in the multivariable model, VPS placement (OR: 27.8, 95% CI [2.2–348.7], P = 0.010) and an increase in the CD4 count of > 100 cells/mL after 24 weeks (OR: 21.9, 95% CI [1.2–408.5], P = 0.039) were independent risk factors for increased CSF protein levels (Table 3).

**Table 3:** Risk factors for raised CSF protein levels in patients with cryptococcal meningitis identified in alogistic regression analysis

Factor	Number	Univariate			Multivariate		
		OR	95%CI	P value	OR	95%CI	P value
Age (years)							
> 50	9	0.6	0.1–4.5	0.657			
≤ 50	77						
Anticryptococcal therapy							
Contained AmB	53	0.4	0.0–3.7	0.405			
Did not contain AmB	34						
HAART regimens							
Missing data	29	1.8	0.4–7.9	0.454			
INSTI	21						
Non-INSTI	37						
VPS placement							
Yes	29	10.9	1.9–64.0	0.008	27.8	2.2–348.7	0.010
No	57						
CSF culture							
Missing data	7						
Positive	54	2.9	0.7–11.7	0.127			
Negative	26						
Blood culture							
Missing data	1						
Positive	28	1.6	0.3–7.6	0.556			
Negative	58						
Increase of CD4 count after 24 weeks(/mL)							
Missing data	45						
> 100	14	8.5	0.9–76.9	0.058	21.9	1.2–408.5	0.039
≤100	28						
Initial CD4 count(/mL)							
Missing data	28						
> 20	26	0.3	0.1–1.4	0.112			
≤ 20	33						
Initial CSF ICH (mmH_2_O)							
Missing data	5						
> 300	52	1.4	0.3–5.9	0.633			
≤ 300	30						
Initial CD4 count (/mL)							
Missing data	28						
> 20	26	0.3	0.1–1.4	0.112			
≤ 20	33						
Initial CSF protein level (g/L)							
Missing data	7						
> 0.5	45	0.8	0.2–3.7	0.801			
≤0.5	35						
Initial CSF WBC (× 10^6^/L)							
Missing data	10						
> 55	64	1.5	0.3–8.4	0.644			
≤ 55	13						
Time of HAART initiation							
Missing data	35						
Before 4 weeks	27	3.6	0.3–38.2	0.282			
After 4 weeks	25						

*AmB* Amphotericin B, *HAART* Highly active antiretroviral therapy, *ICP* intracranial pressure, *INSTI* Integrase strand transfer inhibitor, *VPS* ventriculoperitoneal shunt, *OR* odds ratio, *CI* confidence interval, *ICH* intracranial hypertension

### Complications of VPS placement

Of the 29 patients in the VPS group, one (3.5%) patient died from postoperative infection, nine (31.0%) had transient fever after VPS placement, and one (3.5%) had intestinal perforation.

### Treatment and outcomes

A total of 62.1% (18/29) of the patients in the VPS group and 61.4% (35/57) in the non-VPS group (P = 0.952) were administered an AmB-based regimen. Of the total patients, an integrase strand transfer inhibitor (INSTI)-based regimen was used in 53.8% (14/26) of patients in the VPS group and 21.2% (7/33) of patients in the non-VPS group among patients with accepted available antiviral therapy data (P = 0.009). The initial time of antiviral therapy was 24.0 [11.0–31.0] days in the VPS group and 28.0 [19.0–37.0] days in the non-VPS group after anti-cryptococcal treatment (P = 0.261).

The rate of neuroimaging abnormalities was 14/28 [45.2%] in the VPS group and 20/27 [74.1%] in the non-VPS group before antifungal therapy initiation (P = 0.026). However, the rate of neuroimaging improvement was 16/17 [94.1%] in the VPS group and 2/10 [20.0%] in the non-VPS group (P < 0.001).

**Fig. 3:**
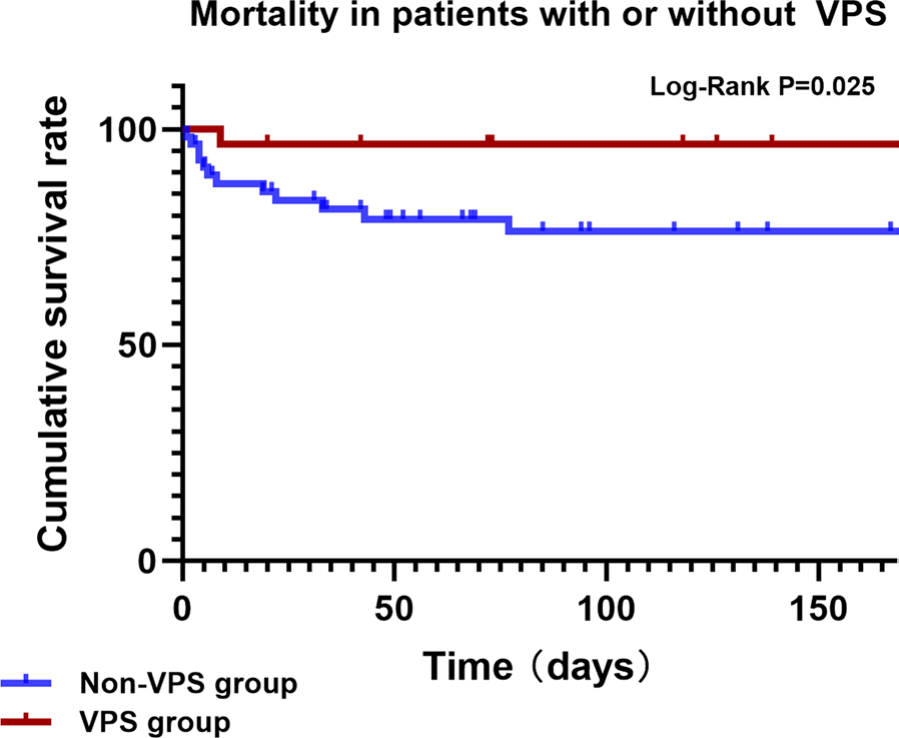
Comparison of cumulative survival rates between the VPS and non-VPS groups (log-rank, P = 0.025)

During the 24-week follow-up, 1 patient in the VPS group died, and 12 patients in the non-VPS group died. The 24-week cumulative survival rate was 83.5% in the non-VPS group and 96.6% in the VPS group (log-rank, P = 0.025; Fig. 3).

Of note, 24.1% (7/29) of patients in the VPS group and 3.5% (2/57) of patients in the non-VPS group were misdiagnosed with tuberculous meningitis and underwent antituberculosis treatment (P = 0.003). In addition, 55.2% (16/29) of the patients in the VPS group and 14% (8/57) of the patients in the non-VPS group used corticosteroids at W24 for the treatment of immune reconstitution inflammatory syndrome (IRIS; P < 0.001).

## Discussion

Although VPS placement is one of the most effective treatments for HCM patients with HICP, its effects on the long-term outcomes of these patients remain unclear. In our present study, we found the following: (1) VPS placement effectively decreased the HICP and mortality of HCM patients; (2) the CSF profiles of HCM patients in the VPS group were profoundly changed (especially increased CSF protein levels and decreased glucose levels); and (3) the frequencies of misdiagnosis with tuberculosis and immune reconstitution inflammatory syndrome were significantly higher in the VPS group than in the non-VPS group.

We observed that the CSF profiles in the VPS group in our study were significantly changed. This is consistent with the findings of previous studies [6, 7, 12]. To date, the underlying mechanisms of the increase in CSF protein content in patients with VPS are unclear. First, a predisposition for “paradoxical” IRIS may have existed. “Paradoxical” IRIS is characterized by initial improvement in clinical manifestations after antifungal therapy followed by deterioration because of HAART-mediated immune restoration in patients with HCM [16], which is similar to our patients’ clinical manifestations. Our study also found that VPS placement and increased CD4 counts were independent risk factors for increased CSF protein levels. Therefore, “paradoxical” IRIS may have triggered changes in clinical presentations and CSF profiles. Second, the placement of a shunting device may have caused increased CSF protein levels. Previous studies [17, 18] have suggested that CSF protein levels are increased by the placement of external drainage devices in patients with Alzheimer’s disease and are associated with trauma resulting from ventricular drain insertion. Therefore, we speculated that the placement of an external drainage device may increase CSF protein levels. Third, the placement of an external drainage device may have stimulated the production of cytokines/chemokines, such as vascular endothelial growth factor, transferrin, and brain-derived protein, in CSF, leading to higher CSF protein levels [17, 19, 20].

Notably, some patients in the VPS group were misdiagnosed with tuberculous meningitis, and some patients were diagnosed with IRIS based on CSF profile changes. A CSF profile similar to that observed in tuberculous meningitis may also be a manifestation of IRIS, and this aspect should be investigated in further studies.

Although one study found that the 10-week survival rate of patients after 1 week of AmB therapy was higher than that after 2 weeks of AmB therapy in an African population [21], our previous study found that the 90-day survival rate of patients treated with AmB for > 14 days was significantly higher than that of patients treated for < 14 days [13]. Wu et al. also found that the duration of AmB-containing treatment during the induction period was a protective factor for better prognoses [22]. Some additional factors, such as CSF WBC, intracranial pressure and CSF glucose, were associated with patient outcomes. Overall, AmB + 5FC was associated with an increased survival rate but was not the sole favorable factor.

This study had some limitations. First, the sample size was small. However, compared with previous studies on VPS, this study included the largest sample size of patients with HCM. Second, the specific mechanisms underlying increased CSF protein levels were not fully investigated. Larger studies focusing on the pathogenesis of increased CSF protein levels after VPS placement are needed. We believe that a comprehensive understanding of the pathogenesis of increased CSF protein levels after VPS placement will improve clinicians’ decisions regarding the management of these patients. Third, our study only included Chinese patients, which may affect the generalizability of our results.

## Conclusions

In conclusion, although VPS placement is effective in controlling intracranial hypertension in HCM patients, it can result in extremely high CSF protein levels and low CSF glucose levels after VPS placement. This could lead to misdiagnosis of tuberculous meningitis and some of them were diagnosed with immune reconstitution inflammatory syndrome. Physicians should be aware of this change in the CSF profiles of HCM patients with VPSs to reduce misdiagnoses and improve long-term prognoses.

## Data Availability

The datasets used and/or analyzed during the current study are included within the article and are available from corresponding author on reasonable request.

## Abbreviations

HIV: Human immunodeficiency virus
CM: Cryptococcal meningitis
HCM: Human immunodeficiency virus-associated cryptococcal meningitis
HICP: High intracranial pressure
VPS: Ventriculoperitoneal shunt
ICP: Intracranial pressure
CSF: Cerebrospinal fluid
HAART: Highly active antiretroviral therapy
BMI: Body mass index
IRIS: Immune reconstitution inflammatory syndrome
OR: Odds ratio
CI: Confidence interval
